# Whole genome sequencing assisted outbreak investigation of Salmonella enteritidis, at a hospital in South Africa, September 2022

**DOI:** 10.1099/acmi.0.000835.v3

**Published:** 2024-11-18

**Authors:** Brian Brümmer, Anthony Marius Smith, Motshabi Modise, Juno Thomas, Hetani Mdose, Ramasedi Mokoena, Dikeledi Baleni

**Affiliations:** 1 Notifiable Medical Conditions, National Institute for Communicable Disease, Johannesburg, South Africa; 2 School of Public Health, University of the Witwatersrand, Johannesburg, South Africa; 3 Centre for Enteric Disease, National Institute of Communicable Disease, Johannesburg, South Africa; 4 Free State Department of Health, Bophelo House, Bloemfontein, South Africa; 5 Community Health, Faculty of Health Science, University of Free State, Bloemfontein, South Africa

**Keywords:** case–control, outbreak, *Salmonella Enteritidis*, whole genome sequencing

## Abstract

Health authorities were notified of a suspected outbreak of foodborne disease in a hospital in South Africa, where staff and patients reported acute onset of abdominal cramps, diarrhoea, fever and rigours after eating a chicken pasta meal. The aim of this report is to discuss the use of whole genome sequencing (WGS) analysis of bacterial isolates to support an epidemiological investigation. An epidemiological investigation led by the Infection Control Manager of the hospital and supported by an outbreak response team was conducted. Standard microbiological procedures were used to process stool samples and culture/identify diarrhoeal pathogens. Bacterial cultures were investigated using WGS performed using Illumina NextSeq technology, and WGS data were analysed using multiple bioinformatics tools, including those available at the Center for Genomic Epidemiology and EnteroBase. Core genome multilocus sequence typing (cgMLST) was used to investigate the phylogeny of isolates. Forty-nine cases were identified, with stool samples collected from 21 cases, and nontyphoidal *Salmonella* isolated from 19 out of 21 (90%) of the samples. All isolates were identified as *Salmonella enterica* serovar Enteritidis and differed from each other by ≤2 allele differences on cgMLST, indicating that isolates are highly genetically related. Delays in testing of food retention samples rendered the negative test results of limited value. A case–control study was conducted; eating chicken pasta was strongly associated with developing gastroenteritis (odds ratio (OR) = 15.4, Chi-Square test with Yates correction *p* value = 0.02). The epidemiological evidence suggests that the chicken pasta was the likely vehicle of transmission in this outbreak, although the source of *S. enterica* serovar Enteritidis remains unknown.

## Data Summary

All WGS data were uploaded to the public EnteroBase platform (http://enterobase.warwick.ac.uk/species/index/senterica) and so are freely available to access at the EnteroBase platform. In addition, sequencing data are deposited in the European Nucleotide Archive under the project accession number PRJEB39546. Individual sample accession numbers are listed in Supplementary Material (available with the online version of this article).

## Introduction

In Africa, foodborne disease (FBD) is considered under-reported [1], yet Africa has the third highest burden of nontyphoidal *Salmonella* [2].

Chicken-type meat can be a predictor of the level of *Salmonella* species found in meat products [3]. Chicken meat is widely consumed; driven by few cultural beliefs against its consumption [4], its higher bio-availability of protein over plant sources [5] and how quickly its production is scaled [6].

Meat production in Africa was low relative to higher income countries, yet with urbanization, and despite avian influenza warnings during 2006 [7], production increased annually by 5% from 2000 to 2011 [4].

More recently, Foot and Mouth disease in South Africa [8], COVID-19 and Ukraine–Russia unrest saw South African meat prices increase, yet chicken prices increased least [9]. This may suggest that South African chicken production may increase further.

With increased poultry production and continued misuse of antimicrobials in humans and animals, drug-resistant strains of *Salmonella* species may surface [10]. The surveillance of such, through whole genome sequencing (WGS) for instance, should be an area of focus.

In 2017, the Centre for Enteric Diseases (CED) of the National Institute for Communicable Diseases in South Africa, implemented WGS analysis of bacterial pathogens, to assist with surveillance and epidemiological investigations of outbreaks. Two concurrent outbreaks during 2018 were linked due to the relatedness of the strains of *Salmonella enterica* serovar Enteritdis demonstrating usefulness of WGS in outbreak investigations [11]. Notably, the largest *Listeria monocytogenes* outbreak was reported in South Africa from 2017 to 2018. WGS was used to identify the source of *L. monocytogenes* [12,13].

The Listeriosis outbreak generated the awareness of FBD in South Africa and improved legislation on the notification of diseases. However, since then foodborne outbreak notifications have not improved and, of the notifications that have been investigated, only 11% are investigated adequately [14,15]; fewer still are published in academic journals.

Publishing outbreak reports and related literature is good practice since it may improve the awareness of FBD and add to the knowledge of those investigating. We report an outbreak of *S. enterica* serovar Enteritidis in the Free-State province, where WGS assisted the investigation. There are lessons learnt to improve food-sample specimen submissions and to improve the quality of data in FBD outbreak investigations in South Africa. This article intends to be part of the precedent to increase the rate of published investigations of outbreaks in South Africa.

## Setting

On 8 September, an astute infection prevention and control (IPC) manager at a hospital in the Mangaung district notified the provincial epidemiologist of a suspected FBD outbreak when the index case, a doctor at that hospital, presented to the emergency department of the same hospital with acute onset abdominal cramps, myalgia, fever, shivers and diarrhoea. The index reported symptoms began 5–7 h after consuming a meal of chicken pasta prepared by the same hospital’s kitchen. The index case further disclosed that four other colleagues, who had shared the meal, were experiencing similar, milder, symptoms. The index case submitted a stool specimen which found *Salmonella* species.

Outbreak investigation was initiated to measure the extent, source and vehicle of transmission of the outbreak. Demographics and clinical characteristics were described. The initial null hypothesis was that the chicken pasta meal prepared by the facility kitchen was associated with developing Salmonellosis. To test the hypothesis, a questionnaire on symptoms and food consumed was sent to doctors on duty on the same day as doctors who tested positive for *Salmonella* species. Evidence generated may assist to identify optimal mitigation measures and recommendations.

## Case summary

A case definition was formed to include any staff member or patient at the facility who reported gastrointestinal symptoms between the 7 and 11 September 2022 and/or eating a lunch meal prepared by the hospital kitchen on the 7 September 2022 and/or having laboratory confirmation of *Salmonella* species. Doctors at the facility had raised clinical suspicion of gastrointestinal symptoms in anyone who may have eaten chicken pasta and reported these to the IPC. Kitchen support staff were line listed by the kitchen manager on Excel. Line lists were collated by the investigating Epidemiologist. We identified 49 cases who met the case definition, of those, 21 stool specimens were collected from public and private laboratories and 19 culture-positive specimens were submitted to the CED. The CED confirmed the identification of bacterial isolates using standard phenotypic microbiological identification and serotyping techniques. Genomic DNA was extracted from cultures and WGS was performed using Illumina NextSeq technology. DNA libraries were prepared using an Illumina Nextera DNA Flex Library Preparation Kit, followed by 2×150 paired-end sequencing runs with ~80 times coverage. Raw sequencing data were uploaded and investigated at the EnteroBase web-based platform (http://enterobase.warwick.ac.uk/species/index/senterica). EnteroBase analysis included serotype confirmation using various genomic serotyping tools and a genomic comparison of isolates based on core genome multilocus sequence typing (cgMLST) data, using the ‘cgMLST V2 + HierCC V1’ scheme. Phylogenetic cluster analysis of cgMLST data was depicted using a GrapeTree-generated minimum spanning tree using the EnteroBase ‘MSTree V2’ algorithm. Five environmental swabs, six food samples and one water sample were analysed with no notable results relating to the outbreak.

## Results

### Line listing

**Fig. 1.**
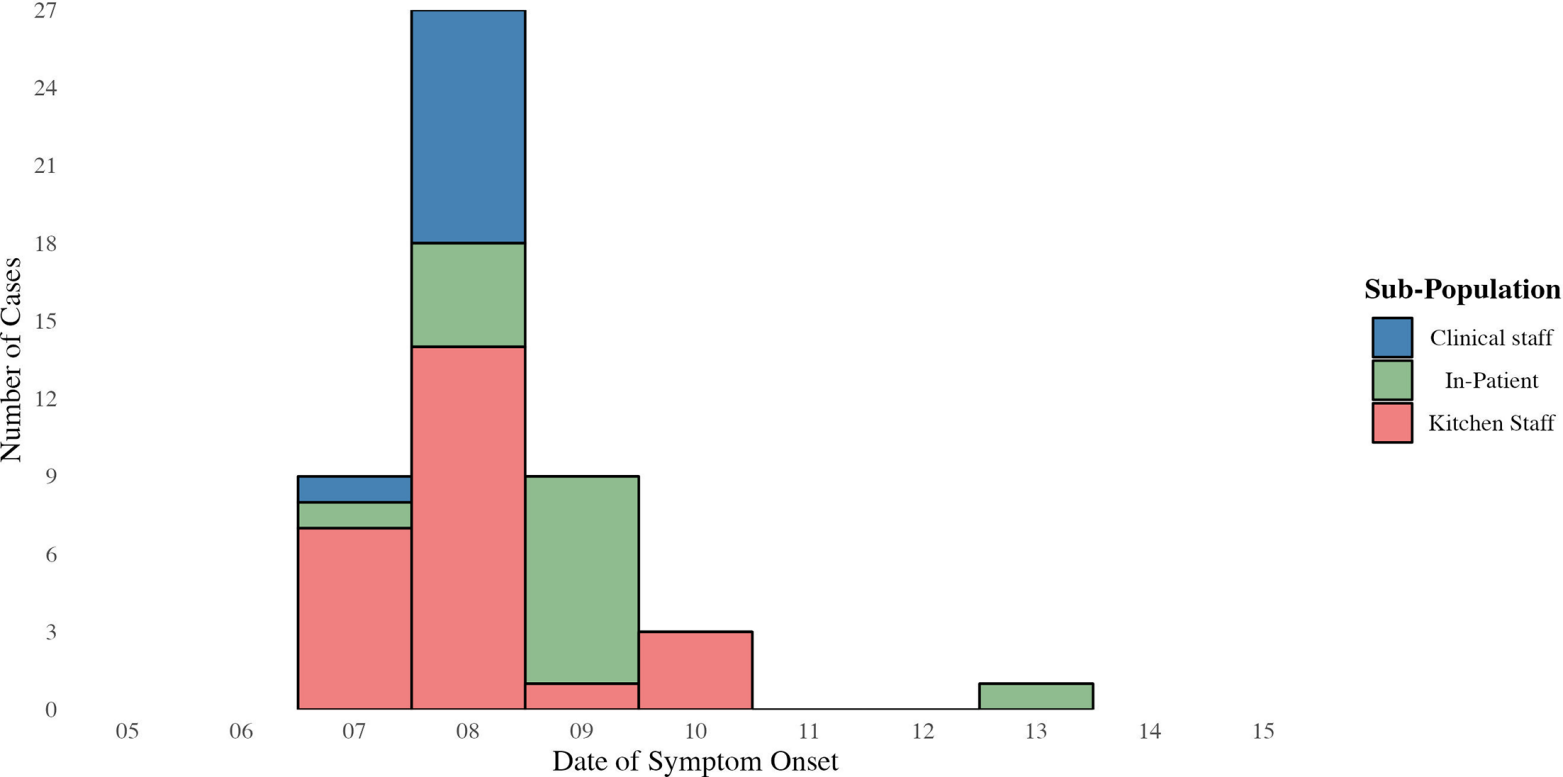
Epidemic curve of all cases (suspected and confirmed) from three sub-populations at the hospital during September 2022.

Descriptive results of the line listing are in Table 1. While the outbreak impacted a large number of kitchen staff (*n* = 24), only one had access to diagnostic. Clinical staff were mainly male compared to the other groups who were mainly female. The most reported symptom was diarrhoea. The epidemic curve (Fig. 1) shows a clear point source transmission pattern. The last case to be reported was an in-patient in a semi-vegetative state – the likely reason for late identification.

**Table 1.** Descriptive table of characteristics of cases identified by the case definition by the type of staff member they were

Variable	Clinical staff, *N* = 10	In-patient, *N* = 14	Kitchen staff, *N* = 25
Epidemiological classification*			
Epidemiological link only	5 (50 %)	1 (7.1 %)	24 (96 %)
Laboratory confirmed	5 (50 %)	13 (93 %)	1 (4.0 %)
**Age†**	45 (35, 58)	58 (41, 65)	36 (32, 40)
Unknown	2	1	0
Sex*			
Female	2 (22 %)	11 (79 %)	20 (80 %)
Male	7 (78 %)	3 (21 %)	5 (20 %)
Unknown	1	0	0
Symptoms*			
Diarrhoea	9 (90 %)	13 (93 %)	23 (92 %)
Abdominal cramps	7 (70 %)	9 (64 %)	19 (76 %)
Loss of appetite	6 (60 %)	10 (71 %)	6 (24 %)
Vomiting	2 (20 %)	5 (36 %)	13 (52 %)
Fever	6 (60 %)	6 (43 %)	8 (32 %)
Muscle pain	6 (60 %)	3 (21 %)	7 (28 %)
Rigours	6 (60 %)	4 (29 %)	1 (4.0 %)
Atechickenpasta*	8 (80 %)	11 (79 %)	25 (100 %)

Unknown variables are omitted from descriptive statistic calculation. An epidemiological link is someone who had suggestive links by person place and time to the outbreak but with no laboratory confirmation. *n (%); †Median (Inter Quartile Range (IQR).

### Environmental

Food retention and water samples revealed no *Salmonella* species. No *Salmonella* species was found from PCR swabs of kitchen surfaces, and drinking water was compliant with South African National Standard (SANS) 241.

The kitchen had adequate hand washing facilities, schedules and training in place. All meals are pre-planned and the hospital has a high turnover with working generators in the event of power failures to the cold storage. The kitchen follows a ‘first-in first-out’ principle.

### Case–control

The doctors who responded were predominantly older males. Full descriptive statistics are in Table 2. There was no one who ate chicken pasta and did not get sick. We therefore added fixed effects of 0.51 to each cell (known as a Haldane–Anscombe Correction [16] so that the OR and upper 95% confidence interval is defined.

**Table 2.** Descriptive statistics of cases and controls of the doctor population

Variable	Overall, *N* = 15	Case, *N* = 8	Control, *N* = 7
Age*	57 (39, 61)	51 (40, 59)	59 (47, 61)
**Sex†**			
Female	2 (13 %)	1 (13 %)	1 (14 %)
Male	13 (87 %)	7 (88 %)	6 (86 %)
**Ate chicken†**			
Yes	6 (40 %)	6 (75 %)	0 (0 %)
No	9 (60 %)	2 (25 %)	7 (100 %)

Controls were recruited through the doctor relations officer at the hospital. Any doctor could responsd if they also ate any of the meals prepared by the kitchen on the day the suspect meal was served. *Median (IQR); †*n* (%).

The OR of association between being a case and exposure to the chicken pasta meal is 15.4, 95% confidence interval (1.21–936.03). The *p*-value of a Chi-Square test with Yates is 0.02 which is sufficient evidence to reject the null hypothesis of OR = 1 at the conventional 0.05 level.

### WGS data analysis

**Fig. 2.**
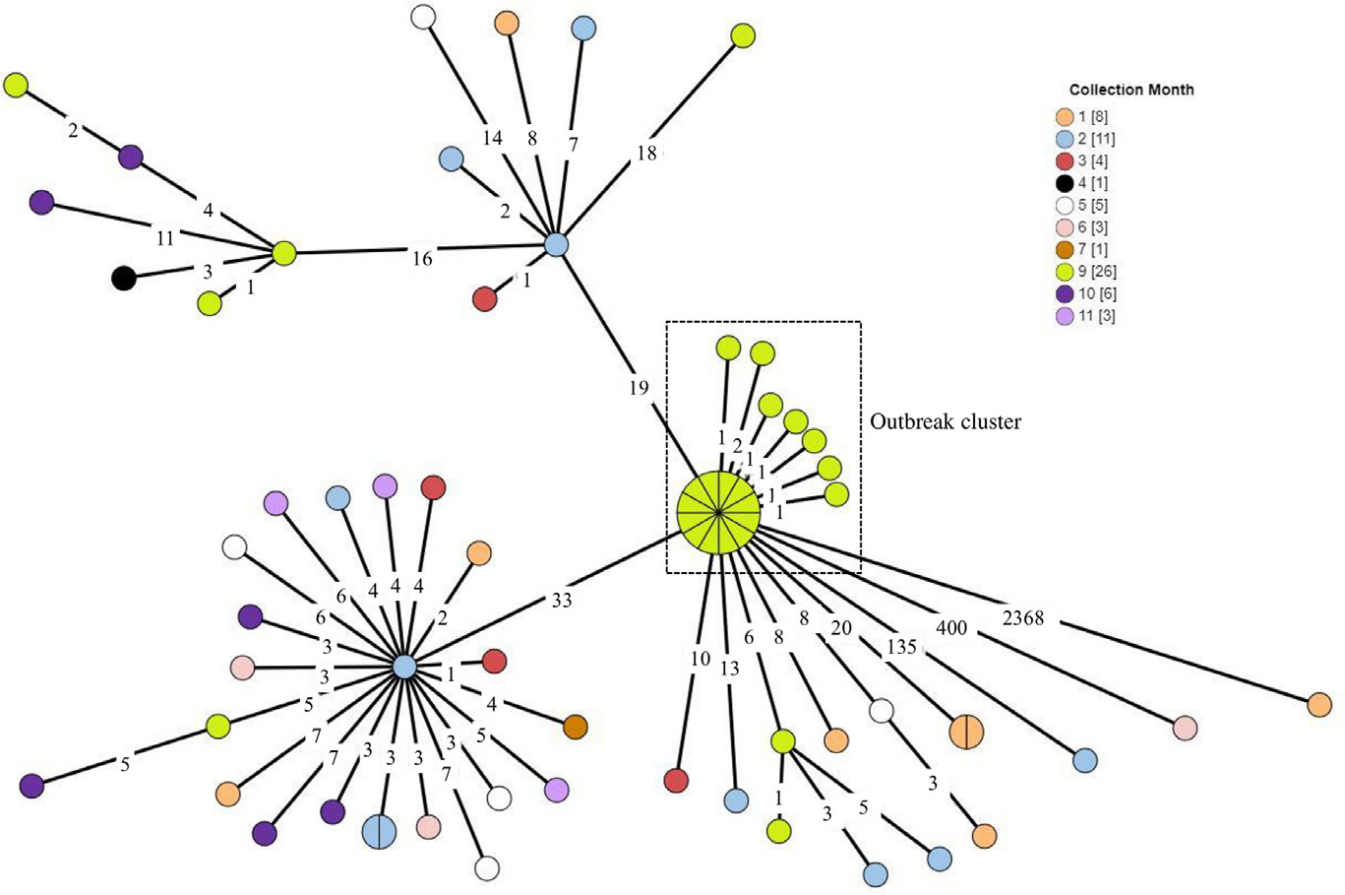
Minimum spanning tree drawn using cgMLST data from *Salmonella* Enteritidis isolates (*n* = 68) sourced from the Free State province of South Africa for the year 2022. The circular nodes represent isolate(s). The larger the circular node, the more isolates which are reflected. The number of segments within a circular node is indicative of the number of isolates. Isolates positioned within the same circular node show no allelic differences when comparing one isolate against another. The number values between adjacent nodes indicate the number of allele differences between connecting nodes (isolates). The outbreak cluster is indicated.

All isolates (*n* = 19) associated with this outbreak were identified as *S. enterica* serovar Enteritidis. WGS data were investigated using cgMLST, which revealed that all isolates clustered together to show ≤2 allele differences when comparing one isolate against another (Fig. 2). Furthermore, 12/19 isolates even showed no allele differences on cgMLST. These all indicated that the cluster of outbreak isolates was highly genetically related and indicative of a high probability of epidemiological relatedness.

## Discussion

The referral of food specimens for testing by the public health laboratory was lacking and did not add to evidence to show the contaminated meal; however, there is strong epidemiological evidence that the chicken was associated with infection. The case–control study showed strong evidence of association between cases and exposure to the chicken pasta. The epidemic curve was highly suggestive of a single-source outbreak. WGS showed cases in the outbreak to be highly genetically related which indicate a single source. South Africa and Free State have frequent power outages; the facility has a serviced generator suggesting the cold storage would have been maintained in case of these power outages. The kitchen uses a first-in first-out principle and has a high patient turnover; therefore, foods are seldom kept in storage for more than 1 week. The kitchen service contract ended at the end of September and a new contractor took over. Many kitchen staff were retained. During refresher training, the new contractor noted that cross-contamination principles in the retained staff were lacking. There is insufficient evidence to identify the source of *S. enterica* serovar Enteritidis; however, we maintain that adequate cooking practices and cross-contamination practices – such as cooking hot enough, and for long enough – substantially reduce the risk of *Salmonella* species in food. We are reminded that *Salmonella* species is common in chicken dishes, and this should be considered when designing the menu for hospital patients.

## Conclusion

We reported an outbreak of *S. enterica* serovar Enteritidis which was associated with a chicken pasta meal and affected 49 patients and found 19 laboratory-confirmed cases were highly genetically related. WGS assisted in confirming this point-source outbreak. Timely referral of food retention samples is necessary to ensure the usefulness of laboratory analysis of referred specimens. Correct food handling and preparation practices remain the mainstay of Salmonellosis prevention. We urge extra attention of adequate cooking time, reducing cross-contamination procedures and hot holding time recommended in future investigations and prevention campaigns. We would add that chicken-containing meals be deliberated over before inclusion in a hospital menu.

## supplementary material

10.1099/acmi.0.000835.v3 Uncited Fig. S1.

## Abbreviations

CED: centre for enteric diseases
cgMLST: core genome multilocus sequence typing
FBD: foodborne disease
IPC: infection prevention and control
OR: odds ratio
WGS: whole genome sequencing
